# Local adaptation with high gene flow: temperature parameters drive adaptation to altitude in the common frog (*Rana temporaria*)

**DOI:** 10.1111/mec.12624

**Published:** 2014-01-20

**Authors:** A P Muir, R Biek, R Thomas, B K Mable

**Affiliations:** * Institute of Biodiversity, Animal Health and Comparative Medicine, University of GlasgowGlasgow, G12 8QQ, UK; † Royal Zoological Society of Scotland, Edinburgh ZooCorstophine Road, Edinburgh, EH12 6TS, UK

**Keywords:** climate, divergent selection, *F*_ST_–*Q*_ST_, phenotypic plasticity

## Abstract

Both environmental and genetic influences can result in phenotypic variation. Quantifying the relative contributions of local adaptation and phenotypic plasticity to phenotypes is key to understanding the effect of environmental variation on populations. Identifying the selective pressures that drive divergence is an important, but often lacking, next step. High gene flow between high- and low-altitude common frog (*Rana temporaria*) breeding sites has previously been demonstrated in Scotland. The aim of this study was to assess whether local adaptation occurs in the face of high gene flow and to identify potential environmental selection pressures that drive adaptation. Phenotypic variation in larval traits was quantified in *R. temporaria* from paired high- and low-altitude sites using three common temperature treatments. Local adaptation was assessed using *Q*_ST_–*F*_ST_ analyses, and quantitative phenotypic divergence was related to environmental parameters using Mantel tests. Although evidence of local adaptation was found for all traits measured, only variation in larval period and growth rate was consistent with adaptation to altitude. Moreover, this was only evident in the three mountains with the highest high-altitude sites. This variation was correlated with mean summer and winter temperatures, suggesting that temperature parameters are potentially strong selective pressures maintaining local adaptation, despite high gene flow.

## Introduction

Observable differences in phenotype are a function of both genetic control and environmental induction (Allendorf & Luikart 2007). Natural selection can act to adapt populations to the local environment, here defined as a fitness advantage of local genotypes over genotypes originating in other environments (Miller *et al.* 2011). However, phenotypic divergence can also be caused by genetic drift (Richter-Boix *et al.* 2010; Lekberg *et al.* 2012) and/or phenotypic plasticity: the ability of a single genotype to produce different phenotypes depending on the environment experienced (Via & Lande 1985). Therefore, observable differentiation between populations in the wild (or lack thereof) does not necessarily indicate local adaptation (Conover & Schultz 1995). Typically, the causes of phenotypic variation are assessed by removing the effect of environment via common garden and/or reciprocal transplant experiments (Hansen *et al.* 2012). However, to understand the adaptive basis of genetic variation, neutral genetic processes (such as genetic drift) must also be accounted for in the observed phenotypic differentiation (Savolainen *et al.* 2007; Hangartner *et al.* 2012).

A common method to account for neutral variation has been to compare population divergence based on quantitative traits (*Q*_ST_) with that based on putatively neutral genetic loci (*F*_ST_) (Whitlock & Guillaume 2009; Lind & Johansson 2011). Comparison of *Q*_ST_ with *F*_ST_ tests whether the quantitative trait divergence is greater than that expected from neutral genetic variation alone (genetic drift) (McKay & Latta 2002). A greater *Q*_ST_ than *F*_ST_ is taken as evidence for divergent natural selection; if *Q*_ST_ equals *F*_ST_, genetic drift alone accounts for observed trait variation; and if *Q*_ST_ is less than *F*_ST_, stabilizing selection is inferred (McKay & Latta 2002; Lind *et al.* 2011). *Q*_ST_ vs. *F*_ST_ analyses, although widely used in evolutionary biology (see Leinonen *et al.* 2008 for a meta-analysis), are the subject of an ongoing debate with regard to their utility as indicators of adaptation (see O'Hara & Merilä 2005; Whitlock & Guillaume 2009; Edelaar *et al.* 2011 for further discussion). Recent adaptation studies have attempted to improve robustness of *Q*_ST_ vs. *F*_ST_ analyses by incorporating the following improvements: (i) contrasting the population pairwise matrices of *Q*_ST_ and *F*_ST_, rather than a single value of *Q*_ST_ and *F*_ST_ averaged across all sites, thereby avoiding biases due to potentially different distributions of the two estimators (Alho *et al.* 2010); (ii) calculating *Q*_ST_ within multiple common environments to avoid genotype-by-environment interactions that can confound comparisons with *F*_ST_ (Gomez-Mestre & Tejedo 2004; Richter-Boix *et al.* 2011; Hangartner *et al.* 2012); (iii) including at least ten populations to reduce the confidence intervals around *Q*_ST_ estimates (Ovaskainen *et al.* 2011); and (iv) using *Q*_ST_ vs. *F*_ST_ analyses as an exploratory tool to identify traits putatively under selection, which can then be used to explore the selective forces acting on phenotypic divergence in more detail (Edelaar & Björklund 2011; Hangartner *et al.* 2012), a step frequently lacking in local adaptation studies (Whitlock 2008).

Local adaptation is typically thought to occur through divergent natural selection acting on isolated populations (Miaud & Merilä 2001). Under this view, high levels of gene flow could swamp the effect of local natural selection through the introduction of maladaptive alleles from differentially adapted populations (Allendorf & Luikart 2007; Bridle & Vines 2007). However, there is increasing empirical evidence that local adaptation also can take place in the face of gene flow (Endler 1977; Richter-Boix *et al.* 2011; Cristescu *et al.* 2012). The level of gene flow required to inhibit local adaptation depends on the strength of selection acting on a trait (Endler 1977). Indeed, it has been suggested that directional selection on important life history traits can maintain divergence between populations at adaptive loci, whilst allowing homogenization in other parts of the genome (Lande 1976; Richter-Boix *et al.* 2011). There is also debate as to whether phenotypic plasticity is itself an adaptive trait, or merely a by-product of fluctuating selection (Via 1993). Phenotypic plasticity can certainly lead to fitness advantages in heterogeneous environments (Lind *et al.* 2011), although the costs of, and limits to, plasticity are poorly understood (Van Buskirk & Steiner 2009). Combining analyses of local adaptation and phenotypic plasticity to draw conclusions about the basis of phenotypic variation in heterogeneous environments can help to elucidate the relative roles of genotype, environment and their interaction (Lind & Johansson 2007).

Species that inhabit heterogeneous environments are subject to spatially varying selection pressures (Miaud & Merilä 2001). Environmental gradients, where parameters vary in a systematic way, are ideal for studying interactions between phenotype and environment (Fukami & Wardle 2005). Altitudinal gradients have been proposed as particularly suitable for studying selection pressures imposed by climatic variables, due to the rapid change in environmental conditions over short geographical distances (Miaud & Merilä 2001). In particular, average temperature has been found to decrease by 6.5 °C for every 1000 m gain in elevation globally (Briggs *et al.* 1997) and acts as a strong selective pressure on ectotherms, due to the direct effect of ambient thermal conditions on physiological processes (Carey & Alexander 2003). Local adaptation to altitude has been observed in a range of ectotherms including insects (e.g. fruit fly; Barker *et al.* 2011), reptiles (e.g. sagebrush lizard; Sears 2005), fish (e.g. redband trout; Narum *et al.* 2013) and amphibians (e.g. wood frog; Berven 1982).

The common frog, *Rana temporaria* (Anura: Ranidae), occurs throughout Europe and is locally adapted to altitude in terms of sexual maturity and UV resistance (Miaud & Merilä 2001), and putatively outlier loci have been identified in relation to elevation in the French Alps (Bonin *et al.* 2006). In Fennoscandia, *R. temporaria* have been well demonstrated to be locally adapted to latitude in a range of larval fitness traits (Laugen *et al.* 2003; Palo *et al.* 2003). Larval fitness and thus size at metamorphosis have consequences for adult survival (Altwegg & Reyer 2003) and are dependent on nongenetic maternal effects (Laugen *et al.* 2002), local adaptation (Hangartner *et al.* 2012) and environment experienced during development (Merilä *et al*. 2000a,b). However, the influence of temperature on larval fitness traits is not fully understood, due to the nonlinear temperature–latitude relationship within the Fennoscandian study area (Laugen *et al.* 2003). In Scotland, *R. temporaria* breed from zero to over a thousand metres above sea level and are the most abundant of only six native amphibian species (Inns 2009). The mountains of Scotland offer replicated altitudinal transects, with a minimum of fragmentation by human activities and continuous habitat suitable for *R. temporaria* (Thompson & Brown 1992; Trivedi *et al.* 2008), avoiding difficulties associated with trying to separate anthropogenic influences and habitat fragmentation from environmental influences. We have previously confirmed the expected linear decline in temperature with altitude in Scotland, but found high levels of gene flow within and between pairs of high- and low-altitude *R. temporaria* breeding sites at a scale of up to 50 km (average pairwise *F*_ST_ = 0.02) and no effect of local temperatures on neutral genetic population structure (Muir *et al.* 2013).

The overall aim of this study was to assess whether local adaptation occurs along altitudinal gradients in the face of high gene flow and to identify potential environmental selection pressures that drive divergent adaptation. Specifically, we aimed to answer the following questions: (i) ‘Do quantitative traits and phenotypic plasticity vary in relation to altitude?’ (ii) ‘Are populations locally adapted by altitude?’ and (iii) ‘What are the environmental drivers of local adaptation by altitude?’

## Methods

### Sampling

Within west central Scotland, five altitudinal gradients were chosen for study based on the presence of known high- and low-altitude *Rana temporaria* breeding sites, mountain height and accessibility (Table 1; see Muir *et al.* 2013 for a map of the sites). The study was set within a limited geographical area (maximum distance between study mountains was 50 km) in order to minimize the effect of latitude and longitude relative to the effect of altitude. Within each of the five mountains, a high-altitude (over 700 m above sea level) and a low-altitude (below 300 m) breeding pool were chosen, giving ten breeding sites in total. Site names refer to the study mountain and whether it is a high- or low-altitude site, for example LOMHIGH.

**Table 1:** Locations of study sites in Scotland including site name (study mountain and whether high or low altitude) with associated abbreviation, latitude, longitude and altitude (metres above sea level)

Site	Abbreviation	Latitude	Longitude	Altitude
Beinn Dubhchraig High	DUBHIGH	56.3951	−4.7506	907
Beinn Dubhchraig Low	DUBLOW	56.4212	−4.6945	198
Beinn Ime High	IMEHIGH	56.2347	−4.8123	921
Beinn Ime Low	IMELOW	56.2046	−4.7628	179
Ben Lawers High	LAWHIGH	56.5423	−4.2291	995
Ben Lawers Low	LAWLOW	56.5002	−4.2354	215
Ben Lomond High	LOMHIGH	56.1857	−4.6478	728
Ben Lomond Low	LOMLOW	56.1598	−4.6363	80
Meall nan Tarmachan High	MNTHIGH	56.5188	−4.2958	900
Meall nan Tarmachan Low	MNTLOW	56.4994	−4.2523	223

For common garden experiments, two-thirds of each of ten separate *R. temporaria* egg masses were collected from each study site during the 2011 breeding season (March–May 2011). Egg masses were defined as a group of eggs within a communal spawning area considered to be from a single mother based on the developmental stage of the eggs and size of the jelly capsules relative to surrounding masses (Griffiths & Raper 1994; Håkansson & Loman 2004). Eggs were collected soon after laying, before having reached Gosner stage 10 (Gosner 1960). Spawn clumps were placed in individual containers filled with source pond water. Spawn was transported immediately back to the laboratory in cool bags, with the aim of keeping the eggs at below 4 °C during transport.

### Quantitative trait variation and phenotypic plasticity in relation to altitude

#### Common garden experiments

On arrival in the laboratory, a subset of ten eggs were removed from each egg mass in order to identify developmental stage and measure egg diameter to the nearest 0.1 mm (Gosner 1960). Egg size has been found to account for some of the variation due to maternal effects in *R. temporaria* (Laugen *et al.* 2002). The remainder of the egg masses were maintained in individual sterilized water tanks at 10 °C until hatching (Gosner stage 22). A randomly selected subset of 30 of the putatively full-sibship tadpoles (Lind & Johansson 2007) was removed from each clump and placed in groups of five in six individual 1.3 L plastic baskets with a 0.1 cm mesh. Two baskets per spawn clump were each placed in temperature treatment rooms, with air temperatures set at 10, 15 and 20 °C, respectively. In total, this gave ten sites*10 families*two baskets (of five tadpoles)*three treatments, which equals 600 replicates. Water quality was maintained using an intermittent flow-through system, where water was slowly added for 2 h every 2 days. Immediately after flow-through, tadpoles were fed *ad libitum* with a 1:2 mixture of finely ground dried fish and rabbit food. The amount of food provided increased with tadpole development to ensure that excess still remained after 2 days. As tadpoles got close to metamorphosis, it became necessary to completely change the water in the tanks once a week to ensure water quality. During complete water changes, water was allowed to adjust to treatment room temperature before tadpoles were added to it and flow-through was kept slow enough that tank temperature did not vary by more than 1.5 °C during cleaning (measured using submerged thermometers). The light regime was maintained at 12-h light/12-h darkness.

At the start of the experiment, at hatching (Gosner stage 22), three tadpoles per spawn clump were measured for snout-vent length (SVL) to the nearest mm and wet weight to the nearest 0.1 g. All tadpoles were allowed to develop until they reached metamorphosis (the end point for the experiment), observed as front leg emergence (Gosner stage 42). SVL and wet weight were measured for all surviving tadpoles. Survival was recorded as the number of tadpoles remaining at the end of the experiment out of the initial number placed in the tanks (tadpoles that died were removed from tanks throughout the experiment). SVL gain and weight at metamorphosis were calculated by subtracting SVL and weight at the beginning of the experiment (using an average per family due to low observed variability in size at hatching; average standard deviation per family: SVL ± 0.3 mm, weight ± <0.1 g) from SVL and weight at the end of the experiment per individual. Larval period was recorded as the number of days from hatching to metamorphosis. Growth rate was calculated as metamorphic weight divided by larval period.

#### Statistical analyses

All statistics were performed in r v2.12.1 (R CoreTeam 2013). To explain the variation in quantitative trait values observed in relation to altitude, a linear mixed model was applied to each trait using the lme4 package (Bates *et al.* 2011). The model consisted of altitude as the fixed factor (as a categorical variable: low or high), with treatment as a fixed factor (10, 15 or 20 °C), basket as a random effect nested within treatment and mountain as a random effect. Each model parameter and interactions were sequentially removed from the model, and a likelihood ratio test was used to evaluate parameter significance. A Tukey's HSD test (Tukey 1953), with associated chi-squared test, was carried out using the final model to evaluate significant differences in pairwise comparisons of means for significant factors.

Phenotypic plasticity was assessed as the ability of a single genotype to show multiple phenotypes in different environments (Merilä *et al*. 2000a). Reaction norms for each site (by mountain and altitude) were plotted for the larval trait mean against the temperature treatment, for each of the quantitative traits. An ancova was carried out using trait as the response variable and temperature and altitude as continuous and discrete predictor variables, respectively, to assess whether the slopes of the reaction norms varied by altitude (low and high).

### Local adaptation in relation to altitude

#### Calculating *Q*_ST_

A linear mixed models approach was used to assess within- and between-site trait variation for the calculation of *Q*_ST_. Site and family were considered as random effects of interest (to be extracted for further calculations), with egg size as a covariate, treatment as a fixed factor and basket as a random factor nested within family. Egg size has been found to account for a large proportion of variation resulting from nongenetic maternal effects in *R. temporaria* (Laugen *et al.* 2002) and inclusion in the model can be used to reduce, but not exclude (Orizaola *et al.* 2013), this as a confounding variable when using wild-collected eggs (Lind & Johansson 2007). Treatment was considered as a fixed factor to account for any variation due to genotype–environment interactions (Laugen *et al.* 2005; Lind & Johansson 2011). Normality of trait distributions was tested using Shapiro–Wilk normality tests. Traits were log-transformed to homogenize variances (as per Hangartner *et al.* 2012). Between-site variance (*V*_b_; variation due to site) and between-family variance (*V*_f_; variation due to family) were extracted from the models as sums of squares. *V*_f_ (due to family) was then converted to *V*_w_ (within-site variance) using the formula *V*_w_ = 3*V*_f_ (Richter-Boix *et al.* 2013). This conservative approach avoids overinflation of *Q*_ST_ by allowing for increased within-family variance that could be a result of the full-sibling design and use of wild eggs (Richter-Boix *et al.* 2013), as multiple paternity (Laurila & Seppa 1998) and clutch piracy (Vieites *et al.* 2004) have been observed in *R. temporaria*. Quantitative trait divergence (*Q*_ST_) values were calculated for each larval trait over all populations and between all population pairs, using the formula *Q*_ST_ trait = *V*_b_/(2*V*_w_ + *V*_b_) (amended from Whitlock 2008; Hangartner *et al.* 2012; Richter-Boix *et al.* 2013).

#### *Q*_ST_–*F*_ST_ comparisons

Global *F*_ST_ (*F*_ST_-G) and pairwise *F*_ST_ between each site (*F*_ST_-P) were previously calculated based on eight microsatellite markers (excluding LOMLOW due to the lack of neutral genetic data; Muir *et al.* 2013; Table S1, Supporting Information); those values are used here for comparison with *Q*_ST_.

Global *Q*_ST_ (*Q*_ST_-G) was first compared with *F*_ST_-G to assess the direction of the relationship within the system as a whole (i.e. whether individuals were under divergent, stabilizing or no selection) and significance was assessed using a Student's *t*-test. Second, a Mantel test (Mantel & Valand 1970) was used to measure dependency between the *F*_ST_ and *Q*_ST_ matrices of site pairwise divergence (*F*_ST_-P and *Q*_ST_-P). Mantel tests were implemented in arlequin v3.5 (Excoffier & Lischer 2010) with 10 000 permutations.

### Environmental drivers of local adaptation to altitude

#### Quantifying environmental parameters in relation to altitude

During the 2010 breeding season, Thermocron i-buttons (Dallas Semiconductor/Maxim, London) were placed at high- and low-altitude breeding sites to record air temperature measurements every 2 h. Data were downloaded to a laptop every 6 months using a USB i-button adapter (Dallas Semiconductor/Maxim) and the software, Thermodata viewer (Thermodata Pty Ltd., Melbourne, Vic, Australia). Dataloggers were removed from the field in October 2011. The water parameters pH (to 0.01 pH), conductivity (to 1 μS/cm) and total dissolved solids (to 1 ppm) were recorded at three points around the edge of each site pool using an HI 98129 Waterproof Tester (Hanna instruments, Leighton Buzzard, UK). Measurements were taken in each season that *R. temporaria* are active (spring, summer and autumn), giving three measurements per site per year. Dissolved oxygen content (to 0.1 mg/L) was recorded during sample collection in spring 2011, at three locations around the edge of each site pool, using a Jenway 9071 portable DO_2_ meter (Jenway, Stone, UK).

Mean annual temperature was calculated by site by averaging the daily mean air temperature. Maximum temperature difference (a measure of environmental heterogeneity) was calculated as the absolute difference between the maximum and minimum temperature recorded per site. For seasonal means, monthly averages were calculated per site and then averaged over March, April and May for spring; June, July and August for summer; September, October and November for autumn; and December, January and February for winter (adapted from Raffel *et al.* 2006; UK Meteorological Office). *R. temporaria* are thought to require temperatures of above 5 °C to induce activity (Laugen *et al.* 2003). Therefore, active period was calculated per site as the number of days per year where the average temperature was at or above 5 °C. Water parameters were recorded as an average per site. Linear regression analysis was used to assess whether each environmental parameter varied predictably with altitude (m).

#### Correlated divergences in adaptive traits and environmental parameters

First, *Q*_ST_ values for traits that showed evidence of local adaptation in relation to altitude, and the mountains where this was observed, were used to create matrices of pairwise divergence between sites. Second, pairwise environmental differences between sites were used to construct environmental divergence matrices for parameters that showed a significant relationship with altitude. Mantel tests were then carried out in arlequin v3.5 using 10 000 permutations to assess the correlation between quantitative trait and environmental divergence. If more than one of the environmental parameter matrices significantly correlated with trait divergence in the Mantel tests, partial Mantel tests were conducted with multiple environmental matrices simultaneously to assess which environmental parameter explained more of the trait divergence and to eliminate any significance biases created by carrying out multiple Mantel tests. A Bonferroni correction was used to assess the significance of the results of the partial Mantel tests.

## Results

### Quantitative trait variation and phenotypic plasticity in relation to altitude

#### Quantitative trait variation

Complete mortality was observed for DUBLOW tadpoles in the 10 °C treatment and for LAWHIGH tadpoles in the 20 °C treatment. Therefore, larval trait data were available for nine populations at 10 and 20 °C and 10 populations at 15 °C (Table 2). Mountain, altitude, treatment and all their interactions significantly changed the log-likelihood when removed from the model for each response variable and were retained in the final models (Table S2, Supporting Information). Based on Tukey's HSD tests, larval period differed significantly between altitudes in all mountains and treatments (Table S3, Supporting Information). However, only DUB, LAW and MNT had consistently shorter larval periods at high than at low altitude in all temperature treatments (Tables 2 and S3, Supporting Information). In contrast, for IME and LOM, the direction of the relationship varied by temperature treatment. Similarly, growth rate was consistently higher at high altitude in DUB, LAW and MNT (5/7 interactions were significant; Table S3, Supporting Information). In contrast, the growth rates in IME and LOM were not significantly different by altitude. Metamorphic weight was only significantly different by altitude for individuals from LAW and MNT at 15 °C (Table S3, Supporting Information). SVL gain was only significantly different by altitude in individuals from one mountain (LOM) and only at 15 and 20 °C. However, for LAWLOW and LOMLOW at 10 °C, quantitative trait values were based on only a single surviving individual (Table 2). High-altitude individuals survived significantly better than low-altitude individuals in all treatments from LOM and MNT, high- vs. low-altitude survival varied by treatment in LAW and IME, and low-altitude individuals from DUB survived significantly better than high-altitude individuals in all treatments (Table S3, Supporting Information).

**Table 2:** Quantitative trait variation by temperature treatment (treatment) and site. Values per site (mean values are shown with their associated standard deviations) are shown for the diameter of *Rana temporaria* eggs at collection (egg size); the weight at metamorphosis minus the weight at hatching (metamorphic weight); the number of days between hatching and metamorphosis (larval period); the gain in snout-vent length (SVL) between hatching and metamorphosis (SVL gain); the increase in weight per day during the larval period (growth rate); and the percentage of tadpoles that survived from hatching to metamorphosis (survival)

Site	Treatment (°C)	Egg size (mm)	Metamorphic weight (g)	Larval period (days)	SVL gain (mm)	Growth rate (mg/day)	Survival (%)
DUBHIGH	10	2.3 ± 0.2	0.8 ± 0.2	111	11.8 ± 0.9	8 ± 2	7
DUBHIGH	15	2.3 ± 0.2	0.8 ± 0.2	39	11.2 ± 0.7	20 ± 4	8
DUBHIGH	20	2.3 ± 0.2	0.3 ± 0.2	22	8.2 ± 1.3	16 ± 9	41
DUBLOW	10	2.2 ± 0.1	NA	NA	NA	NA	0
DUBLOW	15	2.2 ± 0.1	0.6 ± 0.2	76	10.2 ± 1.1	8 ± 2	62
DUBLOW	20	2.2 ± 0.1	0.4 ± 0.1	57	8.8 ± 1.2	7 ± 2	90
IMEHIGH	10	2.1 ± 0.2	0.8 ± 0.2	106	10.5 ± 2.1	8 ± 2	9
IMEHIGH	15	2.1 ± 0.2	0.7 ± 0.2	49	11.4 ± 1.1	14 ± 3	17
IMEHIGH	20	2.1 ± 0.2	0.5 ± 0.1	26	10.3 ± 1.2	19 ± 5	17
IMELOW	10	2.2 ± 0.2	0.8 ± 0.2	121	11.7 ± 0.5	7 ± 2	5
IMELOW	15	2.2 ± 0.2	0.7 ± 0.2	45	10.5 ± 1.0	15 ± 3	39
IMELOW	20	2.2 ± 0.2	0.4 ± 0.1	27	10.4 ± 1.3	15 ± 4	43
LAWHIGH	10	2.2 ± 0.2	0.6 ± 0.1	106	10.8 ± 0.9	6 ± 1	21
LAWHIGH	15	2.2 ± 0.2	0.5 ± 0.2	35	10.1 ± 1.1	11 ± 6	21
LAWHIGH	20	2.2 ± 0.2	NA	NA	NA	NA	0
LAWLOW	10	2.4 ± 0.3	0.6 ± 0.0	137	13.0 ± 0.0	4 ± 0	1
LAWLOW	15	2.4 ± 0.3	0.5 ± 0.1	69	9.8 ± 0.9	7 ± 2	49
LAWLOW	20	2.4 ± 0.3	0.4 ± 0.1	60	8.8 ± 1.0	6 ± 2	53
LOMHIGH	10	2.4 ± 0.1	1.1 ± 0.3	102	10.1 ± 2.0	10 ± 3	10
LOMHIGH	15	2.4 ± 0.1	0.7 ± 0.2	41	8.5 ± 0.9	16 ± 4	26
LOMHIGH	20	2.4 ± 0.1	0.5 ± 0.2	25	6.8 ± 1.6	18 ± 8	37
LOMLOW	10	2.3 ± 0.1	0.6 ± 0.2	43	10.3 ± 1.2	15 ± 3	1
LOMLOW	15	2.3 ± 0.1	0.6 ± 0.0	115	11.9 ± 0.0	5 ± 0	12
LOMLOW	20	2.3 ± 0.1	0.5 ± 0.1	28	10.0 ± 1.2	17 ± 3	16
MNTHIGH	10	2.4 ± 0.2	0.7 ± 0.2	116	11.3 ± 1.5	6 ± 1	28
MNTHIGH	15	2.4 ± 0.2	0.8 ± 0.2	45	9.9 ± 1.0	17 ± 3	75
MNTHIGH	20	2.4 ± 0.2	0.5 ± 0.2	29	9.3 ± 1.1	17 ± 5	44
MNTLOW	10	2.0 ± 0.2	0.7 ± 0.2	127	11.2 ± 1.2	6 ± 1	18
MNTLOW	15	2.0 ± 0.2	0.5 ± 0.1	75	10.1 ± 1.0	6 ± 2	39
MNTLOW	20	2.0 ± 0.2	0.4 ± 0.1	60	9.2 ± 1.4	7 ± 1	39

NA, Quantitative trait data not available due to complete larval mortality.

#### Phenotypic plasticity

**Figure 1:**
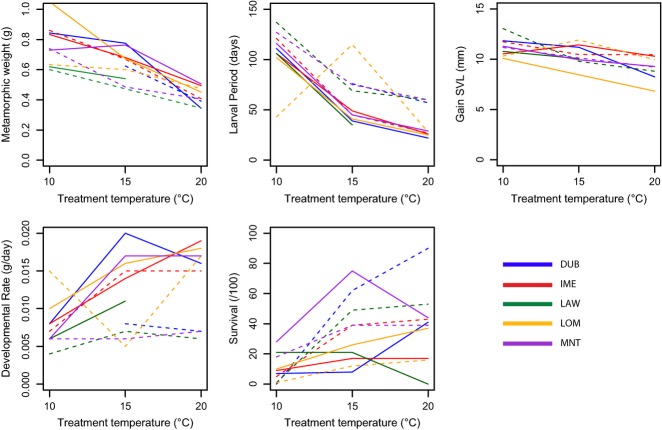
Thermal reaction norms by site for each quantitative trait, demonstrating the relationship between treatment, mountain and altitude. Lines are solid for individuals from high-altitude sites and dashed for individuals from low-altitude sites on each mountain (see Table 1 for mountain name abbreviations). The slope of the line shows the level of phenotypic plasticity at each site (a steeper slope means higher phenotypic plasticity); the location of the line shows the value of the phenotypic trait in relation to other sites (lower down on the graph means a lower trait mean, relative to other sites); if lines are parallel, sites have a similar level of phenotypic plasticity. Values were not available for LAWHIGH at 20 °C and DUBLOW at 10 °C due to complete mortality during the experiment.

Although all sites showed sloping reaction norms for most of the traits measured, the slopes were highly variable (Fig. 1). In general, metamorphic weight decreased with increasing temperature in individuals from all sites (Fig. 1a), and the slope of the reaction norm did not significantly differ between low- and high-altitude individuals (*P* = 0.65, *r*^2^ = 0.43, slope = −0.08). Larval period also decreased with increasing treatment temperature at all sites, except for LOMLOW (Fig. 1b), and there was a significant difference between the slope of the reaction norms for high- and low-altitude individuals (*P* < 0.01, *r*^2^ = 0.72); high-altitude individuals had a steeper reaction norm (slope = −26.22) than low-altitude individuals (slope = −23.42). SVL gain showed only a slight decrease with temperature at all sites, and reaction norms were not different between high- and low-altitude sites (*P* = 0.05, *r*^2^ = 0.25, slope = −0.54; Fig. 1c). Growth rate was higher at 20 °C than at 10 °C for all sites (Fig. 1d). DUB, MNT and LAW had a higher growth rate at high- than at low-altitude sites at all temperatures (high-altitude gained 6 mg/day more than low-altitude individuals, on average; Table S3, Supporting Information). Furthermore, the slope of the reaction norm for growth rate was significantly steeper for individuals from high- (slope = 0.008) than from low-altitude sites (slope = 0.007; *P* < 0.01, *r*^2^ = 0.36). Survival peaked at 15 °C for LAWHIGH and MNTHIGH, but at 20 °C for all other sites (Fig. 1e), and there was a significant effect of altitude on the slope of the reaction norms (*P* < 0.01, *r*^2^ = 0.23, slope = 0.32 and 0.29, respectively), with individuals from high-altitude sites showing a lower increase in survival from the low- to high-temperature treatment than with individuals from low-altitude sites.

### Local adaptation in relation to altitude

*F*_ST_-G across this study system has previously been estimated as 0.02 ± 0.02 (Muir *et al.* 2013). *Q*_ST_-G values were 0.16 ± 0.15 for metamorphic weight, 0.65 ± 0.36 for growth rate, 0.92 ± 0.57 for larval period, 0.49 ± 0.29 for SVL gain and 0.97 ± 0.70 for survival. *Q*_ST_-G values exceeded *F*_ST_-G by at least fivefold and were significantly different for all traits except metamorphic weight (*P* = 0.09, 0.02, 0.03, 0.02 and 0.04, respectively). These results suggest that divergent local adaptation had driven observed phenotypic differentiation between sites in growth rate, larval period, SVL gain and survival. Mantel tests comparing *Q*_ST_-P and *F*_ST_-P (Table 3) showed that *Q*_ST_-P was not significantly explained by *F*_ST_-P (Table 4a) for all traits including metamorphic weight, further (and more robustly) suggesting that quantitative trait variation was not significantly explained by neutral genetic variation and that local adaptation had taken place.

**Table 3:** Comparison of pairwise genetic distances based on *F*_ST_ from microsatellite markers (lower triangle) with *Q*_ST_ of growth rate (upper triangle)

	DUBHIGH	DUBLOW	IMEHIGH	IMELOW	LAWHIGH	LAWLOW	LOMHIGH	MNTHIGH	MNTLOW
DUBHIGH	–	0.423	0.000	0.002	0.054	0.563	0.047	0.000	0.577
DUBLOW	−0.007	–	0.408	0.593	0.121	0.081	0.567	0.407	0.034
IMEHIGH	0.027	0.025	–	0.003	0.052	0.566	0.021	0.000	0.758
IMELOW	0.005	0.000	0.020	–	0.093	0.721	0.017	0.000	0.758
LAWHIGH	0.003	0.001	0.052	0.021	–	0.235	0.118	0.050	0.208
LAWLOW	0.002	0.007	0.037	0.020	−0.012	–	0.682	0.491	0.026
LOMHIGH	0.005	0.019	0.018	0.031	0.012	0.005	–	0.010	0.704
MNTHIGH	0.020	0.025	0.038	0.028	0.022	0.023	0.029	–	0.465
MNTLOW	0.036	0.046	0.070	0.041	0.004	0.004	0.033	0.025	–

**Table 4:** Mantel test results for (a) correlations between quantitative trait divergence (*Q*_ST_-P) for each trait measured and neutral genetic variation (*F*_ST_-P) and (b) quantitative trait divergence (*Q*_ST_-P) and environmental parameters

First matrix	Trait	Second matrix	Mantel's *r*	*P*
(a)				
* Q*_ST-P_	Metamorphic weight	*F*_ST_	0.09	0.33
	Growth rate	*F*_ST_	−0.08	0.66
	Larval period	*F*_ST_	−0.08	0.71
	Snout-vent length Gain	*F*_ST_	0.20	0.16
	Survival	*F*_ST_	0.14	0.28
(b)				
* Q*_ST-P_	Growth rate	Dissolved oxygen content	−0.04	0.56
			Mean annual temperature	0.65	0.06
			Mean spring temperature	0.74	0.01*
			Mean summer temperature	0.83	0.01*
			Mean autumn temperature	0.83	0.01*
			Mean winter temperature	0.87	0.01*
			Active period	0.68	0.07
	Larval period	Dissolved oxygen content	0.14	0.38
		Mean annual temperature	0.47	0.04*
		Mean spring temperature	0.45	0.04*
		Mean summer temperature	0.53	0.02*
		Mean autumn temperature	0.54	0.02*
		Mean winter temperature	0.45	0.05
		Active period	0.55	0.02*

* Significant at *P* < 0.05.

### Environmental drivers of local adaptation to altitude

#### Quantifying environmental parameters in relation to altitude

Temperature data were not available for IMEHIGH due to datalogger failure. The mean annual temperature across all sites was 6.8 ± 2.2 °C, with a 4.5 °C temperature difference on average between high- and low-altitude sites, a maximum recorded temperature of 34.5 °C, a minimum of −18.5 °C and a maximum annual temperature difference of 41.1 ± 6.6 °C (Table 5). Across sites, seasonal means were 6.0 ± 2.1 °C in spring, 11.0 ± 2.5 °C in summer, 5.3 ± 2.5 °C in autumn and −0.5 ± 1.2 °C in winter. Active period varied from 139 to 260 days and pH was neutral to acidic across all sites (Table 5). Conductivity and total dissolved solids showed high variability between sites (39 ± 28 μS; 23 ± 17 ppm), whereas dissolved oxygen content varied little between sites (10.1 ± 1.2 mg/L; Table 5).

**Table 5:** Environmental parameters by site, indicating altitude; temperature parameters: mean annual temperature (°C), maximum temperature difference (maximum − minimum; °C), seasonal mean temperature (spring, summer, autumn and winter; °C) and active period (days); and water parameters: pH, conductivity (μS), total dissolved solids (ppm) and dissolved oxygen content (mg/L). All mean values are accompanied by their standard deviation

Site	Altitude (m)	Annual temp	Temp difference	Spring temp	Summer temp	Autumn temp	Winter temp	Active period	pH	Conductivity	Dissolved solids	Dissolved oxygen
DUBHIGH	900	3.5 ± 6.0	34.5	3.4 ± 1.8	8.5 ± 0.7	2.6 ± 4.6	−2.4 ± 1.3	139	5.9 ± 0.6	16 ± 4	10 ± 3	11.3
DUBLOW	197	8.2 ± 7.3	52.5	7.3 ± 2.7	13.2 ± 0.8	6.6 ± 5.3	−0.4 ± 3.0	225	5.3 ± 1	45 ± 50	30 ± 27	9.8
IMEHIGH*	703	NA	NA	NA	NA	NA	NA	NA	6.0 ± 0.5	41 ± 38	22 ± 18	10
IMELOW	155	9.0 ± 5.2	35.0	8.2 ± 2.2	12.9 ± 0.9	8.4 ± 3.2	2.5 ± 2.3	260	6.3 ± 1	24 ± 9	17 ± 4	8.2
LAWHIGH	990	3.1 ± 4.4	32.5	4.9 ± 1.2	7.8 ± 0.7	2.5 ± 4.6	−2.8 ± 1.5	146	7.0 ± 0.8	26 ± 12	11 ± 4	12.2
LAWLOW	215	7.8 ± 6.1	46.0	6.9 ± 2.6	12.3 ± 0.8	6.2 ± 4.4	0.2 ± 1.9	219	7.1 ± 0.6	108 ± 32	61 ± 14	8.5
LOMHIGH	720	5.4 ± 5.9	43.5	4.2 ± 2.5	9.7 ± 0.6	4.1 ± 4.4	−1.2 ± 1.3	177	4.8 ± 1.1	24 ± 25	12 ± 13	10.6
LOMLOW	77	9.5 ± 6.2	41.0	8.2 ± 2.7	14.1 ± 0.5	8.6 ± 4.0	2.2 ± 1.9	247	6.1 ± 0.7	16 ± 4	10 ± 3	9.3
MNTHIGH	900	4.0 ± 5.5	38.5	3.1 ± 2.9	7.9 ± 0.6	2.4 ± 4.5	−2.6 ± 1.2	152	6.6 ± 0.2	29 ± 19	17 ± 12	10.2
MNTLOW	223	8.0 ± 6.7	46.5	8.0 ± 3.0	13.0 ± 1.5	6.5 ± 4.7	0.4 ± 2.1	231	7.3 ± 0.6	60 ± 14	45 ± 22	10.6

* Temperature data not available due to datalogger failure.

Altitude of site showed a significant regression with dissolved oxygen content (positive association; *r*^2^ = 0.53, *P* < 0.01), mean annual temperature (negative association; *r*^2^ = 0.77, *P* < 0.01), mean seasonal temperature (negative association; spring: *r*^2^ = 0.87, *P* < 0.01; summer: *r*^2^ = 0.98, *P* < 0.01; autumn: *r*^2^ = 0.93, *P* < 0.01; winter: *r*^2^ = 0.82, *P* < 0.01) and active period (negative association; *r*^2^ = 0.95, *P* < 0.01). There was no significant relationship between altitude and pH (*r*^2^ = −0.12, *P* = 0.83), conductivity (*r*^2^ = 0.04, *P* = 0.28), total dissolved solids (*r*^2^ = 0.15, *P* = 0.15) or maximum temperature difference (*r*^2^ = −0.10, *P* = 0.64).

#### Correlated divergences in adaptive traits and environmental parameters

Only growth rate and larval period showed evidence of local adaptation in relation to altitude in individuals from DUB, LAW and MNT (Fig. 1, Tables 4a and S3, Supporting Information) and were used to test for correlated divergences between quantitative traits and environmental parameters. Quantitative trait divergence in growth rate was positively correlated with mean spring temperature, mean summer temperature, mean autumn temperature and mean winter temperature in the Mantel tests (Table 4b). However, only summer and winter temperature remained significantly positively correlated after Bonferroni correction with larval period in the partial Mantel tests (*r* ≥ 0.58, *P* < 0.01; Table S4, Supporting Information). The relative importance of summer and winter temperature in relation to larval period could not be separated as both became nonsignificant after Bonferroni correction when compared in a partial Mantel test (*r* = 0.23, *P* = 0.19 and *r* = 0.51, 0.04, respectively; Table S4, Supporting Information), suggesting that the parameters are related. Quantitative trait divergence in larval period was correlated with between-site divergence in mean annual temperature, mean spring temperature, mean summer temperature, mean autumn temperature and active period in the single Mantel comparisons (Table 4b). However, none of the environmental parameters remained significantly correlated (after Bonferroni correction) with growth rate in the partial mantel tests (Table S4, Supporting Information), suggesting that all the temperature parameters are related.

## Discussion

### Quantitative trait variation and phenotypic plasticity in relation to altitude

#### Quantitative trait variation

The mountains DUB, LAW and MNT had a significantly shorter larval period and a consistently higher growth rate for individuals from high- compared to low-altitude sites in all temperature treatments, suggesting that larval period and growth rate are locally adapted in relation to altitude in these mountains. In contrast, larval period was significantly shorter for IME and LOM high-altitude individuals in two temperature treatments, but longer in the 15 °C treatment, and growth rate was not significantly different, compared to low-altitude individuals (Table S3, Supporting Information). DUB, LAW and MNT are the three highest mountains in this study system (high-altitude sites ≥900 m; IME and LOM high-altitude sites = 703 and 720 m, respectively; Table 1). Therefore, lack of detectable local adaptation to altitude at IME and LOM could be due to the lower absolute elevation from which eggs were collected or the lower relative difference between high- and low-altitude sites. Although geographical distance is well known to limit local adaptation in plants and animals (Galloway & Fenster 2000; Becker *et al.* 2006), this study adds to previous findings of a potential threshold for local adaptation based on environmental parameters (Skelly 2004; Lekberg *et al.* 2012; Richter-Boix *et al.* 2013). Further research into the altitude, or altitudinal difference between sites, at which local adaptation becomes relevant would be interesting for relating environmental conditions to adaptation, particularly in the light of a changing climate.

Differences in larval period between sites at different altitudes and latitudes have often been attributed to a shorter period of growth (activity period) at high altitude/latitude (Lindgren & Laurila 2009a; Orizaola *et al.* 2010). Lower temperatures and shorter growing seasons are thought to favour faster-growing individuals, who can complete metamorphosis before winter dormancy (Lindgren & Laurila 2009b). However, metamorphic weight is an important fitness indicator and a higher metamorphic weight leads to an increased chance of survival as adults (Altwegg & Reyer 2003). Therefore, the higher growth rate observed at the high-altitude sites of DUB, LAW and MNT, in conjunction with a shorter larval period, means that individuals can grow faster without metamorphosing at a smaller size. This is supported by the lack of consistent significant differences in metamorphic weight or SVL gain between high- and low-altitude sites (Table S3, Supporting Information). A positive relationship between latitude and growth rate has been well documented in *Rana temporaria* along latitudinal gradients in Fennoscandia (Merilä *et al*. 2000a,b; Laugen *et al.* 2003). Our results suggest that altitudinal and latitudinal gradients are comparable in their influence on fitness traits and are potentially subject to the same selective pressures.

#### Phenotypic plasticity

All populations showed phenotypic plasticity (the ability of a single genotype to produce different phenotypes depending on the environment experienced; Via & Lande 1985) in terms of metamorphic weight, larval period, growth rate and survival but not SVL gain. However, the slopes of the reaction norms were only significantly different at high- vs. low-altitude sites for growth rate, larval period and survival (*P* < 0.01), with high-altitude individuals showing a greater phenotypic plasticity in all three traits. However, the high variability in reaction norms observed (Fig. 1) led to a poor model fit for both growth rate and survival (*r*^2^ = 0.36 and 0.23, respectively), but not larval period (*r*^2^ = 0.72), suggesting that differences in plasticity between individuals from high- and low-altitude sites are most pronounced in terms of larval period.

The observed greater phenotypic plasticity, higher growth rate and shorter larval period of high- than low-altitude individuals (Fig. 1), resulting in similar weight at metamorphosis across different environments, point to a pattern of countergradient variation. Countergradient variation results in reduced differences in phenotype along an environmental gradient, due to genetic influences counteracting the environmental influences (Conover & Schultz 1995). Countergradient variation has been described in *R. temporaria* along a latitudinal gradient in Scandinavia (Laugen *et al.* 2003) and in response to different pool drying regimes (Richter-Boix *et al.* 2010), with growth rate increasing as time available for development decreases (with increasing latitude and faster pool drying, respectively). As in our study, countergradient variation in relation to altitude in terms of growth rate has been observed in *Rana sylvatica*, and Berven (1982) attributed the countergradient variation pattern to selection acting to minimize the effect of pond temperature on developmental rate.

### Local adaptation in relation to altitude

*Q*_ST_-G exceeded *F*_ST_-G by at least a factor of five on a global scale in all traits except metamorphic weight. Higher *Q*_ST_-G than *F*_ST_-G is interpreted as evidence of divergent selection (i.e. local adaptation; Lind & Johansson 2011). The lack of a significant correlation between *Q*_ST_-P and *F*_ST_-P when considered pairwise by site (Table 4a) also suggested that quantitative trait variation cannot be explained by neutral genetic variation alone and thus that populations are locally adapted. Correlations between *Q*_ST_-P and *F*_ST_-P matrices are thought to give more robust results regarding the presence of local adaptation than comparisons of global values (Hangartner *et al.* 2012), and we found that both the traditional approach of comparing global values and the approach of comparing pairwise values gave evidence of local adaptation in larval period, growth rate, SVL gain and survival. However, metamorphic weight only showed evidence of local adaptation when using the pairwise correlation, suggesting a lower level of adaptation in this trait. Of the larval traits measured, only growth rate and larval period were consistent in the direction of the difference in trait means between high and low altitude and only in the three mountains with the highest high-altitude sites (DUB, LAW and MNT). Therefore, although there is evidence for local adaptation in all fitness traits, only growth rate and larval period appear to be locally adapted specifically to altitude.

*F*_ST_ estimates calculated from microsatellites have been criticized as they can result in downwardly biased values due to their high polymorphism (Edelaar *et al.* 2011; Meirmans & Hedrick 2011). Reduced *F*_ST_ values can thus lead to an incorrect conclusion that local adaptation has taken place when compared with *Q*_ST_ values (Edelaar *et al.* 2011). However, Muir *et al*. (2013) compared genetic distance calculated using *F*_ST_ for this system with that calculated using Jost's D (Jost 2008) and found them to be comparable, suggesting that the genetic distance estimator is robust in this study. Early environment exposure can also bias results when using wild eggs, leading to inflated *Q*_ST_ values. We cannot account for this source of bias in our study, but given that our results show a *Q*_ST_ of at least five times higher than *F*_ST_ in the traits identified as locally adapted and are significantly different, we are confident that the conclusion that local adaptation has taken place is robust. Our results suggest that local adaptation has occurred within altitudinal gradients in Scotland despite the previous finding of extensive gene flow and limited population structure (Muir *et al.* 2013). High levels of gene flow are generally thought to inhibit local adaptation between sites by introducing alleles that are adapted to other locations and potentially maladaptive in the new location (North *et al.* 2010). However, local adaptation in the face of high gene flow has also been observed in *R. temporaria* in Sweden in response to varying pond canopy cover (Richter-Boix *et al.* 2010) and different pond drying regimes (Lind *et al.* 2011). As the level of gene flow that will inhibit local adaptation depends on the strength of the local selective force (Richter-Boix *et al.* 2010), the local adaptation to altitude of *R. temporaria* in Scotland, in the face of high gene flow, suggests that strong selective pressures are driving trait differentiation.

### Environmental drivers of local adaptation to altitude

Between-site divergence in growth rate showed a significant correlation with mean winter temperature and mean summer temperature (*r* > 0.70, *P* < 0.01 and *r* > 0.5, *P* < 0.01, respectively; Table S4, Supporting Information). Larval period showed a significant correlation with all the temperature parameters assessed (annual and seasonal means and active period; Table 4) and their relative importance could not be separated (Table S4, Supporting Information). These results suggest that temperature parameters are important selective forces driving local adaptation by altitude, with lower temperatures potentially selecting for a higher growth rate and shorter larval period. A higher growth rate is likely to increase survival during colder winters, due to additional storage of reserves prior to overwintering. Therefore, individuals with a lower growth rate will be selected against in colder winter environments. Similarly, storing more resources in a shorter period of time, even when temperatures experienced during larval development are cooler, will allow completion of metamorphosis prior to overwintering and thus increased survival. The mechanism that facilitates higher growth rates in high- compared to low-latitude/altitude individuals has been suggested to be increased feeding activity due to decreased predator presence in colder environments (Urban 2010; Torres-Dowdall *et al.* 2012).

## Conclusion

Variation in temperature provides a strong environmental selection pressure, with temperature parameters influencing local adaptation even in the face of high gene flow in *Rana temporaria*. Temperature is set to rise within the west of Scotland between 0.8 and 4.4 °C in the next 50 years (depending on emissions scenario and uncertainty range; Kundzewicz *et al.* 2007). Therefore, ongoing global warming has the potential to cause fitness changes in populations of *R. temporaria*. Further research is needed to identify why only the highest mountains show local adaptation, and whether absolute temperature or temperature difference between sites is driving divergence, in order to further elucidate the relationship between temperature changes and fitness.

## Data accessibility

Sample locations, environmental data, phenotypic trait data and R-scripts submitted to DRYAD: doi:10.5061/dryad.r95c6.

Microsatellite data: DRYAD entry doi:10.5061/dryad.kh466.

## Supporting Information

Table S1 Summary of microsatellite genotyping results from Muir *et al*. (2013), including number of samples per site (*n*), allelic richness (*A*_r_), observed heterozygosity (*H*_o_) and expected heterozygosity (*H*_e_). **Table S2** Results of the likelihood ratio test to evaluate parameter significance showing the trait of interest (Trait), the model used (Model), the degrees of freedom within the model (d.f.), the log-likelihood of the model (Loglik) and the probability that the alternative model is different from the null model (p). **Table S3** Results of Tukey's HSD test of significant difference between the means of low- and high-altitude sites, by mountain and treatment, per trait. **Table S4** Partial Mantel test results for correlations between quantitative trait divergence (*Q*_ST_-P) and environmental parameters that showed a significant correlation in the Mantel tests.
