# Supplementary material for: Local adaptation with high gene flow: temperature parameters drive adaptation to altitude in the common frog (Rana temporaria)
Source: Mol Ecol. 2014 Jan 20;23(3):561–74. doi: 10.1111/mec.12624 (PMC4285318; doi:10.1111/mec.12624)
Supplement: Table S1 — Summary of microsatellite genotyping results from Muir et al. (2013), including number of samples per site (n), allelic richness (Ar), observed heterozygosity (Ho) and expected heterozygosity (He). Table S2 Results of the likelihood ratio test to evaluate parameter significance showing the trait of interest (Trait), the model used (Model), the degrees of freedom within the model (d.f.), the log-likelihood of the model (Loglik) and the probability that the alternative model is different from the null model (p). Table S3 Results of Tukey's HSD test of significant difference between the means of low- and high-altitude sites, by mountain and treatment, per trait. Table S4 Partial Mantel test results for correlations between quantitative trait divergence (QST-P) and environmental parameters that showed a significant correlation in the Mantel tests. [file mec0023-0561-SD1.doc]

*Table S1: Summary of microsatellite genotyping results from Muir et al. (2013), including number of samples per site (n), allelic richness (A_r_), observed heterozygosity (H_o_) and expected heterozygosity (H_e_). Standard deviations are indicated for mean values; H_o_ lies within the standard deviation of H_e_ at all sites.*

| Site | n | A_R_ | H_o_ | H_e_ |
| --- | --- | --- | --- | --- |
| DUBHIGH | 30 | 5.17 ± 0.33 | 0.64 ±0.2 | 0.62 ± 0.22 |
| DUBLOW | 28 | 5.83 ± 0.44 | 0.66 ± 0.21 | 0.62 ± 0.23 |
| IMEHIGH | 30 | 5.22 ± 0.43 | 0.57 ± 0.21 | 0.59 ± 0.21 |
| IMELOW | 40 | 5.06 ± 0.41 | 0.63 ± 0.21 | 0.62 ± 0.22 |
| LAWHIGH | 26 | 5.62 ± 0.19 | 0.7 ± 0.19 | 0.64 ± 0.2 |
| LAWLOW | 17 | 4.88 ± 0 | 0.74 ± 0.2 | 0.63 ± 0.2 |
| LOMHIGH | 30 | 5.78 ± 0.44 | 0.59 ± 0.22 | 0.63 ± 0.21 |
| LOMLOW* | 0 | NA | NA | NA |
| MNTHIGH | 21 | 6.54 ± 0.32 | 0.68 ± 0.16 | 0.66 ± 0.17 |
| MNTLOW | 32 | 7.63 ± 0.86 | 0.7 ± 0.15 | 0.72 ± 0.14 |

*Genetic data unavailable

Table S2: Results of the likelihood ratio test to evaluate parameter significance showing: the trait of interest (Trait), the model used (Model); the degrees of freedom within the model (Df); the log likelihood of the model (Loglik); and the probability that the alternative model is different from the null model (p). A significantly higher log likelihood indicates a better fit of the model to the data.

| Trait | Model | Df | Loglik | p |
| --- | --- | --- | --- | --- |
| Larval period | Mountain*Altitude*Treatment/Basket | 55 | 21250.3 |  |
|  | Mountain*Altitude | 11 | -3546.2 | <0.01* |
|  | Mountain | 6 | -3600.3 | <0.01* |
|  | Altitude*Treatment/Basket | 13 | -2881.7 | <0.01* |
|  | Altitude | 3 | -3637.1 | <0.01* |
|  | Mountain*Treatment/Basket | 31 | -2962.6 | <0.01* |
|  | Treatment/Basket | 7 | -3174.1 | <0.01* |
| Growth rate | Mountain*Altitude*Treatment/Basket | 55 | 3220.2 |  |
|  | Mountain*Altitude | 11 | 3025.4 | <0.01* |
|  | Mountain | 6 | 2890.9 | <0.01* |
|  | Altitude*Treatment/Basket | 13 | 3031.3 | <0.01* |
|  | Altitude | 3 | 2910.4 | <0.01* |
|  | Mountain*Treatment/Basket | 31 | 2969.9 | <0.01* |
|  | Treatment/Basket | 7 | 2858.2 | <0.01* |
| Metamorphic weight | Mountain*Altitude*Treatment/Basket | 55 | 431.7 |  |
|  | Mountain*Altitude | 11 | 173.2 | <0.01* |
|  | Mountain | 6 | 128.1 | <0.01* |
|  | Altitude*Treatment/Basket | 13 | 330.3 | <0.01* |
|  | Altitude | 3 | 135.9 | <0.01* |
|  | Mountain*Treatment/Basket | 31 | 358.3 | <0.01* |
|  | Treatment/Basket | 7 | 295.8 | <0.01* |
| SVL gain | Mountain*Altitude*Treatment/Basket | 55 | -1184.9 |  |
|  | Mountain*Altitude | 11 | -1341.2 | <0.01* |
|  | Mountain | 6 | -1392.8 | <0.01* |
|  | Altitude*Treatment/Basket | 13 | -1334.2 | <0.01* |
|  | Altitude | 3 | -1450.4 | <0.01* |
|  | Mountain*Treatment/Basket | 31 | -1256.0 | <0.01* |
|  | Treatment/Basket | 7 | -1352.5 | <0.01* |
| Survival | Mountain*Altitude*Treatment/Basket | 55 | 24651.5 |  |
|  | Mountain*Altitude | 11 | 541.7 | <0.01* |
|  | Mountain | 6 | 186.8 | <0.01* |
|  | Altitude*Treatment/Basket | 13 | 162.4 | <0.01* |
|  | Altitude | 3 | 47.8 | <0.01* |
|  | Mountain*Treatment/Basket | 31 | 446.4 | <0.01* |
|  | Treatment/Basket | 7 | 109.2 | <0.01* |

*significant at p<0.05

Table S3: Results of Tukey’s HSD test of significant difference between the means of low- and high-altitude sites, by mountain and treatment, per trait. A positive difference between the means shows that individuals from low-altitude have a greater mean trait value than those from high-altitude, and a negative difference between the means shows that individuals from high-altitude have a greater mean trait value than those from low-altitude.

| Trait | Mountain | Treatment | Difference between means | Lower 95% Confidence Interval | Upper 95% Confidence Interval | p |
| --- | --- | --- | --- | --- | --- | --- |
| Larval period (days) | DUB | 10°C | NA | NA | NA | NA |
|  | DUB | 15°C | 37.00 | 37.00 | 37.00 | 0.00* |
|  | DUB | 20°C | 35.00 | 35.00 | 35.00 | 0.00* |
|  | IME | 10°C | 15.00 | 15.00 | 15.00 | 0.00* |
|  | IME | 15°C | -4.00 | -4.00 | -4.00 | 0.00* |
|  | IME | 20°C | 1.00 | 1.00 | 1.00 | 0.00* |
|  | LAW | 10°C | 31.00 | 31.00 | 31.00 | 0.00* |
|  | LAW | 15°C | 34.00 | 34.00 | 34.00 | 0.00* |
|  | LAW | 20°C | NA | NA | NA | NA |
|  | LOM | 10°C | 13.00 | 13.00 | 13.00 | 0.00* |
|  | LOM | 15°C | 2.00 | 2.00 | 2.00 | 0.00* |
|  | LOM | 20°C | 3.00 | 3.00 | 3.00 | 0.00* |
|  | MNT | 10°C | 11.00 | 11.00 | 11.00 | 0.00* |
|  | MNT | 15°C | 30.00 | 30.00 | 30.00 | 0.00* |
|  | MNT | 20°C | 31.00 | 31.00 | 31.00 | 0.00* |
| Growth rate (g/day) | DUB | 10°C | NA | NA | NA | NA |
|  | DUB | 15°C | -0.01 | -0.02 | -0.01 | 0.00* |
|  | DUB | 20°C | -0.01 | -0.01 | -0.01 | 0.00* |
|  | IME | 10°C | 0.00 | -0.01 | 0.01 | 1.00 |
|  | IME | 15°C | 0.00 | 0.00 | 0.01 | 1.00 |
|  | IME | 20°C | 0.00 | -0.01 | 0.00 | 0.11 |
|  | LAW | 10°C | 0.00 | -0.02 | 0.01 | 1.00 |
|  | LAW | 15°C | -0.01 | -0.02 | -0.01 | 0.00* |
|  | LAW | 20°C | NA | NA | NA | NA |
|  | LOM | 10°C | 0.00 | -0.02 | 0.01 | 1.00 |
|  | LOM | 15°C | 0.00 | -0.01 | 0.00 | 1.00 |
|  | LOM | 20°C | 0.00 | -0.01 | 0.00 | 1.00 |
|  | MNT | 10°C | 0.00 | -0.01 | 0.00 | 1.00 |
|  | MNT | 15°C | -0.01 | -0.01 | -0.01 | 0.00* |
|  | MNT | 20°C | -0.01 | -0.01 | -0.01 | 0.00* |
| Metamorphic weight (g) | DUB | 10°C | NA | NA | NA | NA |
|  | DUB | 15°C | -0.15 | -0.36 | 0.06 | 0.66 |
|  | DUB | 20°C | 0.05 | -0.06 | 0.15 | 1.00 |
|  | IME | 10°C | 0.01 | -0.29 | 0.31 | 1.00 |
|  | IME | 15°C | -0.01 | -0.17 | 0.16 | 1.00 |
|  | IME | 20°C | -0.08 | -0.24 | 0.08 | 0.98 |
|  | LAW | 10°C | 0.00 | -0.55 | 0.56 | 1.00 |
|  | LAW | 15°C | -0.17 | -0.32 | -0.02 | 0.01* |
|  | LAW | 20°C | NA | NA | NA | NA |
|  | LOM | 10°C | -0.39 | -0.94 | 0.19 | 0.77 |
|  | LOM | 15°C | -0.04 | -0.23 | 0.15 | 1.00 |
|  | LOM | 20°C | 0.02 | -0.15 | 0.18 | 1.00 |
|  | MNT | 10°C | 0.01 | -0.15 | 0.17 | 1.00 |
|  | MNT | 15°C | -0.28 | -0.40 | -0.16 | 0.00* |
|  | MNT | 20°C | -0.10 | -0.22 | 0.02 | 0.30 |
| SVL gain (mm) | DUB | 10°C | NA | NA | NA | NA |
|  | DUB | 15°C | -0.95 | -2.65 | 0.76 | 0.96 |
|  | DUB | 20°C | 0.54 | -0.30 | 1.38 | 0.83 |
|  | IME | 10°C | 1.20 | -1.25 | 3.65 | 0.99 |
|  | IME | 15°C | -0.95 | -2.29 | 0.38 | 0.64 |
|  | IME | 20°C | 0.17 | -1.11 | 1.45 | 1.00 |
|  | LAW | 10°C | 2.27 | -2.22 | 6.77 | 0.99 |
|  | LAW | 15°C | -0.55 | -1.77 | 0.68 | 1.00 |
|  | LAW | 20°C | NA | NA | NA | NA |
|  | LOM | 10°C | 1.83 | -2.78 | 6.44 | 1.00 |
|  | LOM | 15°C | 1.86 | 0.33 | 3.39 | 0.00* |
|  | LOM | 20°C | 3.28 | 1.95 | 4.60 | 0.00* |
|  | MNT | 10°C | -0.13 | -1.46 | 1.20 | 1.00 |
|  | MNT | 15°C | 0.18 | -0.81 | 1.16 | 1.00 |
|  | MNT | 20°C | -0.06 | -1.03 | 0.90 | 1.00 |
| Survival (%) | DUB | 10°C | NA | NA | NA | NA |
|  | DUB | 15°C | 0.54 | 0.54 | 0.54 | 0.00* |
|  | DUB | 20°C | 0.49 | -0.49 | 0.49 | 0.00* |
|  | IME | 10°C | -0.04 | -0.04 | -0.04 | 0.00* |
|  | IME | 15°C | 0.22 | 0.22 | 0.22 | 0.00* |
|  | IME | 20°C | 0.26 | 0.26 | 0.26 | 0.00* |
|  | LAW | 10°C | -0.20 | -0.20 | -0.20 | 0.00* |
|  | LAW | 15°C | 0.28 | 0.28 | 0.28 | 0.00* |
|  | LAW | 20°C | NA | NA | NA | NA |
|  | LOM | 10°C | -0.09 | -0.09 | -0.09 | 0.00* |
|  | LOM | 15°C | -0.14 | -0.14 | -0.14 | 0.00* |
|  | LOM | 20°C | -0.21 | -0.21 | -0.21 | 0.00* |
|  | MNT | 10°C | -0.10 | -0.10 | -0.10 | 0.00* |
|  | MNT | 15°C | -0.36 | -0.36 | -0.36 | 0.00* |
|  | MNT | 20°C | -0.05 | -0.05 | -0.05 | 0.00* |

*significant at p<0.05

NA: Quantitative trait data not available due to complete larval mortality

Table S4: Partial Mantel test results for correlations between quantitative trait divergence (Q_ST_-P) and environmental parameters that showed significant correlation in the Mantel tests.

| First matrix | Trait | Second matrix | Mantel's r | p | Third matrix | Mantel's r | p |
| --- | --- | --- | --- | --- | --- | --- | --- |
| Q_ST_ | Growth rate | Mean Spring Temperature | 0.12 | 0.32 | Mean Summer Temperature | 0.58 | <0.01* |
|  |  | Mean Spring Temperature | 0.22 | 0.23 | Mean Autumn Temperature | 0.58 | 0.04 |
|  |  | Mean Spring Temperature | 0.12 | 0.36 | Mean Winter Temperature | 0.70 | <0.01* |
|  |  | Mean Summer Temperature | 0.42 | 0.01* | Mean Autumn Temperature | 0.41 | 0.13 |
|  |  | Mean Summer Temperature | 0.23 | 0.19 | Mean Winter Temperature | 0.51 | 0.04 |
|  |  | Mean Autumn Temperature | 0.11 | 0.34 | Mean Winter Temperature | 0.50 | 0.02 |
| Q_ST_ | Larval period | Mean Annual Temperature | 0.32 | 0.10 | Mean Spring Temperature | 0.27 | 0.16 |
|  |  | Mean Annual Temperature | 0.24 | 0.15 | Mean Summer Temperature | 0.35 | 0.04 |
|  |  | Mean Annual Temperature | <0.01 | 0.45 | Mean Autumn Temperature | 0.31 | 0.13 |
|  |  | Mean Annual Temperature | 0.12 | 0.35 | Active Period | 0.01 | 0.42 |
|  |  | Mean Spring Temperature | 0.01 | 0.49 | Mean Summer Temperature | 0.31 | 0.12 |
|  |  | Mean Spring Temperature | 0.02 | 0.52 | Mean Autumn Temperature | 0.35 | 0.11 |
|  |  | Mean Spring Temperature | 0.22 | 0.23 | Active Period | 0.26 | 0.13 |
|  |  | Mean Summer Temperature | 0.13 | 0.28 | Mean Autumn Temperature | 0.22 | 0.18 |
|  |  | Mean Summer Temperature | 0.34 | 0.02* | Active Period | 0.21 | 0.17 |
|  |  | Mean Autumn Temperature | 0.35 | 0.05 | Active Period | 0.00 | 0.70 |

*significant after Bonferroni correction (p≤0.01)
